# Biosynthesis of *trans-*4-hydroxyproline by recombinant strains of *Corynebacterium glutamicum* and *Escherichia coli*

**DOI:** 10.1186/1472-6750-14-44

**Published:** 2014-05-19

**Authors:** Yulan Yi, Huakai Sheng, Zhimin Li, Qin Ye

**Affiliations:** 1 State Key Laboratory of Bioreactor Engineering, East China University of Science and Technology, 130 Meilong Road, Shanghai 200237, China; 2 Shanghai Collaborative Innovation Center for Biomanufacturing Technology, Shanghai, China

**Keywords:** *Trans*-4-hydroxy-L-proline, Recombinant *Corynebacterium glutamicum*, Recombinant *Escherichia coli*, Proline 4-hydroxylases

## Abstract

**Background:**

*Trans-*4-hydroxy-L-proline (*trans*-Hyp), one of the hydroxyproline (Hyp) isomers, is a useful chiral building block in the production of many pharmaceuticals. Although there are some natural biosynthetic pathways of *trans*-Hyp existing in microorganisms, the yield is still too low to be scaled up for industrial applications. Until now the production of *trans*-Hyp is mainly from the acid hydrolysis of collagen. Due to the increasing environmental concerns on those severe chemical processes and complicated downstream separation, it is essential to explore some environment-friendly processes such as constructing new recombinant strains to develop efficient process for *trans*-Hyp production.

**Result:**

In this study, the genes of *trans-*proline 4-hydroxylase (*trans-*P4H) from diverse resources were cloned and expressed in *Corynebacterium glutamicum* and *Escherichia coli*, respectively. The *trans*-Hyp production by these recombinant strains was investigated. The results showed that all the genes from different resources had been expressed actively. Both the recombinant *C. glutamicum* and *E. coli* strains could produce *trans*-Hyp in the absence of proline and 2-oxoglutarate.

**Conclusions:**

The whole cell microbial systems for *trans*-Hyp production have been successfully constructed by introducing *trans-*P4H into *C. glutamicum* and *E. coli*. Although the highest yield was obtained in recombinant *E. coli*, using recombinant *C. glutamicum* strains to produce *trans*-Hyp was a new attempt.

## Background

Hydroxyproline (Hyp) is a specific amino acid component of collagen. The amount of Hyps varies from 80 to 100 residues per 1000 residues in mammalian collagen, which can be used to estimate collagen content and act as an important indicator to collagen quality [1]. There are five naturally occurring Hyps, including three diastereomers of 4-hydroxyproline and two diastereomers of 3-hydroxyproline. Among them, *trans*-4-hydroxy-L-proline is the most abundant component in the constitution of collagen and can enhance the procollagen synthesis. Its derivative N-acetyl *trans*-4-hydroxyproline (oxaceprol) is an atypical inhibitor of inflammation and useful for the treatment of diseases affecting the connective tissues such as osteoarthritis [2]. *Trans*-Hyp has been widely used in medicine, biochemistry, food, cosmetic and other aspects of industry [3]. Additionally, *trans*-Hyp has also been found in the composition of some secondary metabolites such as actinomycins and echinocandins [4].

*Trans*-Hyp is manufactured industrially most by acid hydrolysis of mammalian collagen because of its rich amount in the collagen. However, it obviously results in many environmental issues and brings great difficulties into the down stream processing [5]. There are several identified pathways of hydroxyproline biosynthesis. In animal tissue, 4-hydroxyproline is catalyzed by prolyl 4-hydroxylase, which takes peptidyl proline as substrate rather than free proline [6]. 4-hydroxy-2-oxoglutaric acid can be enzymatically transformed to hydroxyproline [7]. Some bacteria or fungi have been found to form hydroxyproline via fermentation directly [8]. Although the titer of product is low, these findings show the possibility of utilizing biological processes to produce *trans*-Hyp.

The proline 4-hydroxylases (P4Hs) have been identified from several microbial strains, which can catalyze the hydroxylation of L-proline at the 4-position to produce *trans*-Hyp in the presence of 2-oxoglutarate, oxygen and ferrous ion [9-11]. P4Hs have an optimum pH range of 6.0 to 7.5 and temperature range of 30°C to 40°C. Its activity is inhibited by metal ions such as Zn^2+^ and Cu^2+^. Lawrence et al. have studied the effect of co-substrates on the hydroxylation of L-proline by P4H and pointed that 2-oxoglutarate was essential for proline hydroxylation since the replacement of 2-oxoglutarate with 2-oxopentanoate, 2-oxoadipate, pyruvate or 2-oxomalonate (all at 0.5 mM) led to no detectable hydroxylation of L-proline [10]. Although 2-oxoglutarate as the oxygen donator is required for hydroxylation of L-proline to 4-hydroxy-L-proline in vitro, it is unnecessary to add extra 2-oxoglutarate in vivo in the production of 4-hydroxy-L-proline by recombinant strains. 2-oxoglutarate is a key metabolic intermediate in the tricarboxylic acid cycle (TCA cycle) in *Escherichia coli* strain, which can result in the formation of hydroxyproline from glucose and proline directly [12]. Shibasaki et al. have analyzed the possible metabolic pathways of 2-oxoglutatate. They concluded that 2-oxoglutatate can be supplied either through the action of proline dehydrogenase (PutA) from L-proline or through the action of isocitric dehydrogenase (Icd) from glucose. The addition of L-proline to a glucose-containing minimal medium had a positive effect on both the proline 4-hydroxylase activity and production level [13]. But the availability of intracellular proline may still be limited because the biosynthesis of proline in wild type *E. coli* is strictly regulated to very low level. Thus, the precursor and co-factor in the microbial production of hydroxyproline need to be considered simultaneously.

*Corynebacterium glutamicum* is one of the most important industrial microorganisms and widely used in amino acids, vitamins and nucleic acids production [14]. Leuchtenberger et al. has summarized the commercial application of *C. glutamicum* to the fermentative production of amino acids [15]. Lee et al. has reported a novel glutamate and proline producing method through the utilization of phenol in *C. glutamicum*[16]*.* Masaaki Wachi reported a strategy for optimizing the industrial production of amino acids by reinforcing the export systems of *C. glutamicum*[17]*.* The metabolic pathways of amino acids are sophisticated and controlled tightly in *C. glutamicum.* But *C. glutamicum* as the platform of amino acid production has been studied in details and there are lots of molecular tools used for its genetic modifications, which contribute to *C. glutamicum* as one of the most popular host systems [18,19]. Additionally, Ikeda et al. [20] and Kalinowski et al. [21] have completed genome sequencing of *C. glutamicum* ATCC13032, which made *C. glutamicum* into a new era of system biology. To overproduce amino acids by *C. glutamicum*, not only the modification of biosynthetic pathway and regulation mechanism but also the transportation of amino acids plays significant roles in the final yield of a particular amino acid [22,23].

**Figure 1:**
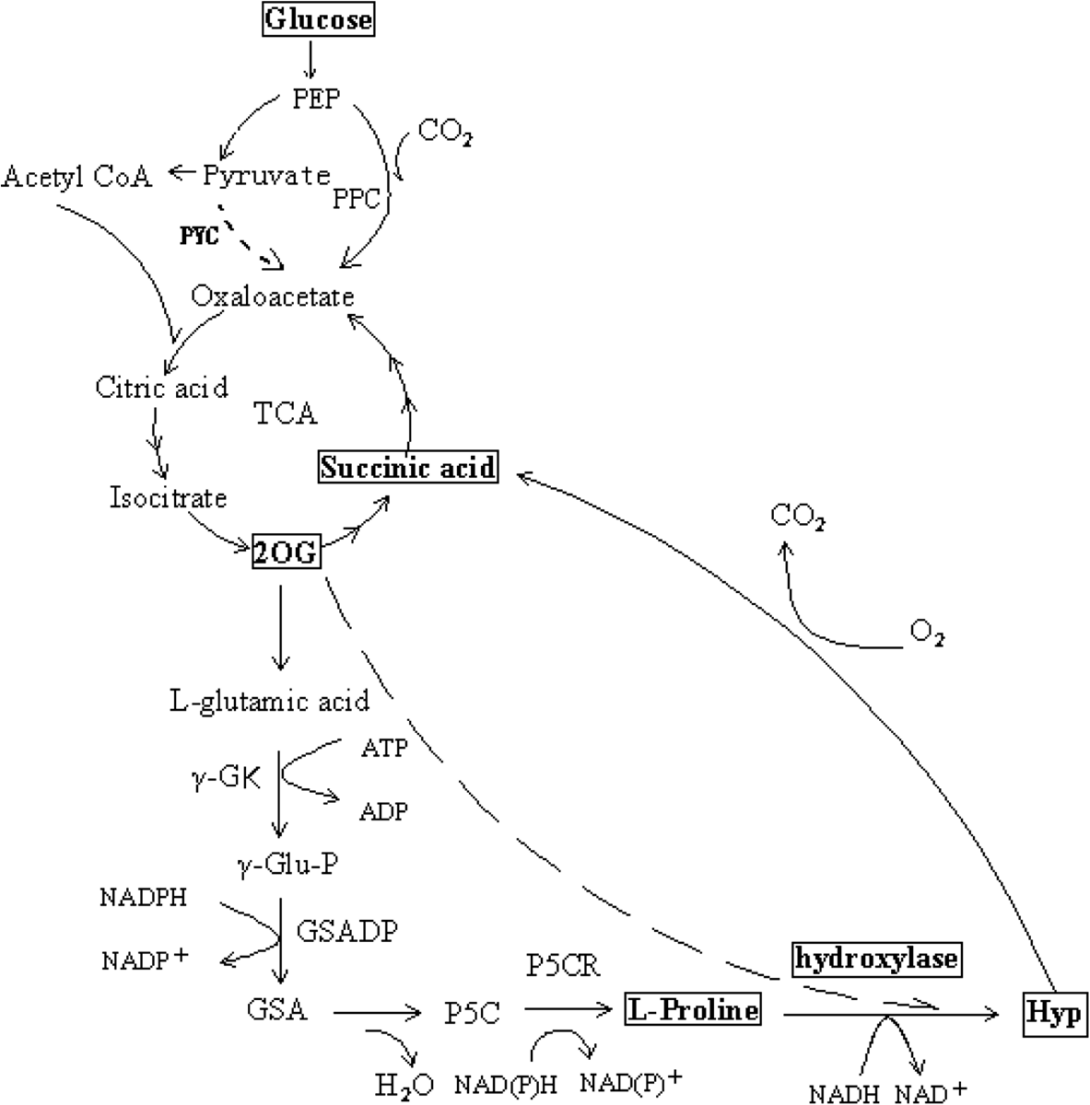
**The metabolism of *trans*-4-hydroxyproline in recombinant bacteria.** 2OG: 2 - 2-Ketoglutaric acid; γ-GK: γ- Glutamyl kinase; γ-Glu-P: γ- Glutamyl phosphate; GSADH; Glutamyl phosphate reductase; GSA: Glutamyl semialdehyde; P5C: Pyrroline - 5 - carboxylic acid; P5CR: P5C reductase; Hyp: Hydroxyproline.

To produce *trans*-Hyp by microbial fermentation, it is essential to contain both the proline pathway and subsequent hydroxylation activity in the microbial cells. Considering the metabolic pathway in *C. glutamicum* or *E. coli* (Figure 1), proline and 2-oxoglutarate can be produced from glucose. NADPH and ATP can also be regenerated during the glucose metabolism. Although there are some efforts being conducted in *E. coli*, for example, Hyp was produced from glucose using a recombinant *E. coli* by introducing a proline 4-hydroxylase and the mutated *proB* (encodingγ-glutamyl kinase) gene encoding the feedback resistant enzyme [24]. However, the development of recombinant *C. glutamicum* for hydroxyproline secretion has never been reported. In this study, genes of *trans-*P4H from diverse sources were screened and cloned into different *C. glutamicum* strains. The recombinant *E. coli* expressed different genes were also investigated. Meanwhile, a surprisingly high yield in shake flasks by recombinant *E. coli* without extra addition of L-proline was achieved.

## Results and discussion

### Construction of *trans*-Hyp producing recombinant strains

There are several genes being speculated as putative L-proline 4-hydroxylase gene in the database, including genes in *Pseudomonas stutzeri* ***,*** *Janthinobacterium* sp*., Bordetella bronchiseptica RB50, Bradyrhizobium japonicum, Achromobacter xylosoxidans C54* and *Dactylosporangium*. sp. Using PCR, we cloned and obtained the putative genes of P4H from *P. stutzer* and *B. bronchiseptica RB50*, named *p4hP* and *p4hB.* They were ligated to the corresponding plasmids after digestion and converted to *C. glutamicum* and *E. coli*, respectively. The length of *p4hP* was 918 bps while *p4hB* was 924 bps. These sequences were 100% identical to the reported genes in NCBI. The gene of *trans*-P4H from *Dactylosporangium sp.* (*p4hD*) had been expressed in *E. coli* successfully and can transform L-proline with good enzymatic properties [11-13,24-26]. The length of *p4hD* was 816 bps encoding a 272-amino-acid polypeptide with the molecular weight of 29,715 daltons [11,26]. In this study, *p4hD* was applied with some modifications on the nuclear bases. The original gene sequence of *p4hD* was analyzed (http://www.kazusa.or.jp/codon/) and the results showed there were some rare codons for both *C. glutamicum* and *E. coli.* It has been reported that rare codons are strongly associated with low level of protein expression [27]. Codon optimization for heterologous protein expression has often been shown to drastically increase protein expression [28]. Thus, the rare codons of *p4hD* gene were substituted for those used with high frequency in *C. glutamicum* and the GC content was adjusted from 73% to 61% through synonymous conversion, which was close to that of *C glutamicum*. The modified gene of *p4hD* was synthesized according to the above modifications (Additional file 1).

**Figure 2:**
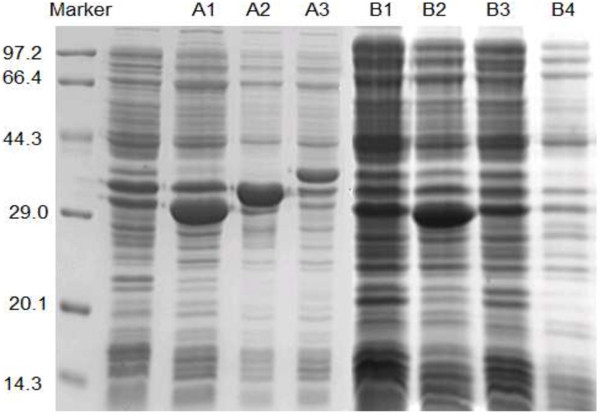
**Expression of *trans*-P4Hs in different strains.** A1: *E. coli* BL21/pET28a-*p4hD*; A2: *E. coli* BL21/pET28a-*p4hP*; A3: *E. coli* BL21/pET28a-*p4hB*. B1: *C. glutamicum* ATCC15940/pECXK99E-*p4hD*; B2: *C. glutamicum* ATCC21355/pECXK99E-*p4hD*; B3: *C. glutamicum* ATCC21157/pECXK99E-*p4hD*; B4: *C. glutamicum* 49-1/ pECXK99E- *p4hD*.

The expression of P4H is one of the important aspects on the construction of trans-Hyp biosynthetic pathway. Figure 2 shows the SDS-PAGE of *trans*-P4Hs expressed in recombinant *C. glutamicum* and *E. coli.* All the recombinant *trans*-P4Hs were expressed as soluble proteins without inclusion bodies. It was obviously that the recombinant *trans-*P4Hs in *E. coli* were expressed much more than those in *C. glutamicum* (Figure 2)*.* Many factors have influences on the expression of foreign proteins including promoters, the host-vector system and cultural conditions etc. [29,30]. Since it was the first to express *trans-*P4Hs in *C. glutamicum*, more comprehensive studies such as promoter selection and culture condition optimization will be considered in our future work.

### Comparison of P4H activities

Oxygenases are widely applied in industry since they can catalyze the highly specific oxyfunctionalization of unactivated C-H bonds under mild conditions, especially transferring molecular oxygen to a substrate [31]. P4H belongs to a family of 2-oxoacid-dependent dioxygenase, which is a monomeric protein and utilizes the monomeric rather than polymeric substrates [10]. The activities of *trans-*P4Hs using recombinant whole cells in this study were measured (Table 1). Our data indicated that the expressed protein level and enzymatic activity was higher at 30°C. The results also showed that the plasmids were very stable as the plasmid stabilities of recombinant *E. coli* and *C. glutamicum* strains were all more than 98% at the end of fermentation.

**Table 1:** **Comparison of *trans *-P4Hs activities and *trans *-Hyp production by different recombinant *C. glutamicum *** **and *E. coli *** **strains**

**Strains**	**Specific activities (U/mg · wet cell weight)**	***Trans*-Hyp (g/L)**	OD600 (OD620)*
*C. glutamicum* ATCC13032/pEC-XK99E- *p4hD*	37.4 ± 1.4	0.072 ± 0.001	5.5 ± 0.7
*C. glutamicum* ATCC13032/pEC-XK99E- *p4hP*	20.7 ± 1.1	0.106 ± 0.002	7.3 ± 0.5
*C. glutamicum* ATCC13032/pEC-XK99E- *p4hB*	40.7 ± 0.8	0.079 ± 0.016	5.4 ± 0.03
*C. glutamicum* ATCC15940/pEC-XK99E- *p4hD*	12.9 ± 0.5	0.103 ± 0.001	14.0 ± 0.2
*C. glutamicum* ATCC21355/pEC-XK99E- *p4hD*	35.9 ± 0.1	0.087 ± 0.005	6.6 ± 0.2
*C. glutamicum* ATCC21157/pEC-XK99E- *p4hD*	12.3 ± 0.9	0.112 ± 0.004	13.3 ± 0.1
*C. glutamicum* 49-1/pEC-XK99E- *p4hD*	12.4 ± 0.6	0.113 ± 0.001	13.8 ± 0.5
*E. coli* BL21/pET-28a -*p4hD*	60.4 ± 1.8	0.470 ± 0.028	6.5 ± 0.2
*E. coli* BL21/pET-28a -*p4hP*	22.2 ± 0.5	0.126 ± 0.007	7.3 ± 0.05
*E. coli* BL21/pET-28a -*p4hB*	50.0 ± 2.2	0.115 ± 0.006	6.9 ± 0.1

*Optical density at 600 nm for *E. coli* and 620 nm for *C. glutamicum.*

The recombinant cells with expressing of different genes showed different levels of catalytic activities toward L-proline. The activity of *trans-*P4H expressed by *E. coli* BL21/ pET28a-*p4hD* was the highest among all the constructed recombinant strains. The new cloned and expressed genes from *P. stutzeri* and *B. bronchiseptica* also showed interested activities. As for different host strains, *E. coli* represented better than *C. glutamicum*, which may be related to the performance of corresponding plasmid. Four L-proline producing strains of *C. glutamicum* were used as expression host strains and the resulted recombinant strains showed different enzymatic activities. The highest specific enzymatic activity among *C. glutamicum* strains was 40.7 U/mg · wet cell by *C. glutamicum* ATCC13032/pEC-XK99E-*p4hB*. However, the specific enzymatic activity of recombinant *E. coli*/pET28a -*p4hD* was up to 60.4 U/mg · wet cell. The growth of three recombinant *E. coli* strains was similar. But there was significant difference among the recombinant *C. glutamicum* strains. The recombinant *C. glutamicum* strains with higher specific enzymatic activities grew less than those with lower specific enzymatic activities. Additionally, the enzymatic activity of *E. coli* BL21 /pET28a -*p4hD* was similar to that of *E. coli* W1485/pWFH1 and higher than that of *E. coli* BL21/pET24-*p4h*1 of [12,13]. The *p4hD* in *E. coli* W1485/pWFH1 was the original one in *Dactylosporangium* sp*.,* while *p4hD* in *E. coli* BL21/pET24-*p4h*1 was modified. Although the codon optimization in this study was designed for *C. glutamicum*, the results indicated that it was also successfully in *E. coli.*

### *Trans*-Hyp production in flasks

The production of *trans-*Hyp by different recombinant *C. glutamicum* and *E. coli* strains was also shown in Table 1. The yields of *trans-*Hyp by these recombinant strains depended both on the enzymatic activity of P4H and cell growth. *E. coli* BL21/ pET28a-*p4hD* had the highest yield, which was coincided of its specific enzymatic activity. Although the recombinant *E. coli* strains grew similarly in the production medium, there was significant difference in the production of *trans-*Hyp which did not keep the same level with the specific enzymatic activities. The productions of *trans-*Hyp by recombinant *C. glutamicum* strains were also much less than that of *E. coli* BL21/pET28a-*p4hD.* It was due to both the less expression of *trans-*P4H and less cell growth in *C. glutamicum*. The L-proline production of four *C. glutamicum* strains was also less than 1 g/L. There was little difference of *trans-*Hyp production among the recombinant strains of *C. glutamicum* with same gene *p4hD*, despite that some strains had better enzymatic performance and proline production.

Using the recombinant strains to directly synthesize *trans-*Hyp from glucose via fermentation was achieved since both the enzymes and precursors needed in the process were available. The over expressed foreign *trans-*P4Hs catalyzed the hydroxylation of L-proline at the *trans*-4 position, while 2– ketoglutarate was supplied by glucose through TCA cycle and then oxidatively decarboxylated to succinate (Figure 1). It was reported that proline was demanded in the production of Hyp by recombinant *E. coli* only with *p4h* gene. The carbon in proline added during the fermentation only flowed into amino acids synthesized from TCA cycle intermediates and not into gluconeogenesis [13]. However, the accumulated Hyp was at a relative high level even without the addition of proline in this study. It could be understood that *Corynebacterium* had the powerful biosynthetic pathway of proline [32,33]. The biosynthetic pathway of proline has also been identified in *E. coli*, which may contribute to the synthesis of *trans*-Hyp by recombinant *E. coli* strains. The modification of proline pathway in *E. coli* enhanced the yield of Hyp, whereas the formation of Hyp can also relieve the feedback inhibition of proline [24]. The amount of proline (0-4 mM) promoted the production of *trans-*Hyp. However, continuously addition of L-proline didn’t improve the production yield significantly (Table 2). In this study, the time of cultivation was significantly less than those reported in the literatures, which might attribute to the different media used and also indicated there was great potential with optimization. In fact, 2.28 g/L of *trans-*Hyp was produced by recombinant *E. coli* without adding L-proline in flasks with a little modification of media and 6.72 g/L was achieved by supplement only 4 mM L-proline.

**Table 2:** Hyp production under different L-proline supplementation

**Supplementary addition of L- proline (mM)**	**0**	**1**	**2**	**4**	**8**	**12**
Hyp (g/L)	2.28	2.81	3.25	6.72	5.56	6.32

In order to further increase the biosynthesis of *trans*-Hyp by recombinant *C. glutamicum* and *E. coli*, alternative approaches should be considered as well. In *E. coli*, the degradation of proline should be overcome. Although the *trans*-Hyp production by a *putA* mutant of *E. coli* was not improved furthermore, the yield based on the proline utilized was enhanced greatly. In both *C. glutamicum* and *E. coli*, the expression of recombinant P4H as one of the oxygenases is involved in the physiological metabolism of host cells including the cofactor, co-substrate and oxygen. Moreover, without a powerful proline synthetic pathway in *E. coli,* the availability and transportation of substrate will limit the transformation seriously.

## Conclusions

In this study, two new and a modified *trans*-P4Hs were expressed in *C. glutamicum* and *E. coli* successfully. Different amount of *trans-*Hyp were produced by these recombinant strains detected. Although the yield in recombinant *C. glutamicum* was less than that in recombinant *E. coli*, *C. glutamicum* as a native proline producing strain was worthy of further optimizing. This is the first report of producing *trans-*Hyp by introducing L-proline 4-hydroxylase into *C. glutamicum.*

## Methods

### Strains and plasmids

The bacterial strains and plasmids used in this study are listed in Table 3. *E. coli* BL21(DE3) and five *C. glutamicum* strains among which four were reported to produce L-proline were used as hosts. Plasmids pET-28a and pEC-XK99E were applied as the vectors respectively. *Trans-* 4-hydroxy-L-proline was purchased from Sigma-Aldrich Trading Co., Ltd.

**Table 3:** Strains and plasmids used in this study

**Strains & plasmids**	**Properties**	**Source/reference**
*E. coli*		
JM109	Cloning host	Our laboratory
BL21(DE3)	*F-, ompT, hsdS(rBB-mB-), gal, dcm (DE3)*	Our laboratory
*C. glutamicum*		
ATCC13032	Wild-type	Our laboratory
ATCC 15940	L-Proline production	Our laboratory
ATCC 21355	L-Proline production	[27]
ATCC 21157	L-Proline production	[27]
49-1 Plasmids	L-Proline production	Our laboratory
pET-28a	His_4_-tag, T7 promoter, Kan^r^	Our laboratory
pEC-XK99E	*E. coli* - *C. glutamicum* shuttle expression vector, Kan^r^	Our laboratory
pET-28a-*p4hP*	pET-28a containing the *p4h* gene from *P. stutzeri*	This study
pEC-XK99E-*p4hP*	pEC-XK99E containing the *p4h* gene from *P. stutzeri*	This study
pET-28a-*p4hB*	pET-28a containing the *p4h* gene from *B. bronchiseptica*	This study
pEC-XK99E-*p4hB*	pEC-XK99E containing the *p4h* gene from *B. bronchiseptica*	This study
pET-28a- *p4hD*	pET-28a containing the *p4h* gene from *Dactylosporangium* sp.	This study
pEC-XK99E-*p4hD*	pEC-XK99E containing the *p4h* gene from *Dactylosporangium* sp.	This study

^r^ indicates resistant.

### Construction of recombinant strains

The full sequences of *p4h* encoding predicted hydroxylases were amplified from the strains’ genomic DNA as followed in Table 4. The primers used in this study for gene cloning and plasmid construction were also as listed in Table 4, which were incorporated *Sal*I/*EcoR*I restriction sites for recombinant *E. coli* and *Sal*I/*Xba*I for recombinant *C. glutamicum*. The PCR products were digested with restriction enzymes above and inserted into the vector pET-28a and pEC-XK99E respectively, which resulted into the expression plasmid pET-28a-*p4h* and pEC-XK99E-*p4h*. The inserted fragment was sequenced and verified the identity to the anticipated sequence. Recombinant plasmid was transferred into *E. coli* competent cells by chemical CaCl_2_ method. The preparation of *C. glutamicum* competent cell and electro transformation of exogenous gene were conducted according to the method in reference [34].

**Table 4:** Primers used in this study for gene cloning and plasmid construction

**Primer name**	**Sequences (5’ → 3’)**	**Source**
*p4hP* –pET-28a-S	TGTAGAATTCATGATCTCACCTGCGCA	*P. stutzeri*
*p4hP* –pET-28a-A	ATATAAGCTTCTAGCTGCCGACCAGCTTC	
*p4hP* -pEC-XK99E-S	TAATGAATTCGTGAACCCTATGCAAGC	*P. stutzeri*
*p4hP* -pEC-XK99E -A	ATAGTCTAGATCAGAGATACTGTTGCGG	
*p4hB*–pET-28a-S	TGATGAATTCCTAGCGCTCGACCAGTTT	*B. bronchiseptica*
*p4hB* –pET-28a-A	CTGCAAGCTTATGATTTCACCTGCTCAGG	
*p4hB* -pEC-XK99E-S	TATAGAATTCATGATCTCACCTGCCCAG	*B. bronchiseptica*
*p4hB* -pEC-XK99E -A	TGTCTCTAGATTATTCCACCAGCTTCAG	
*p4hD* –pET-28a-S	TATAGAGCTCATGCTGACTCCGACCGA	*Dactylosporangium* sp.
*p4hD* –pET-28a-A	GATCAAGCTTTTAAACTGGCTGGGCAAG	
*p4hD* -pEC-XK99E-S	TATAGAGCTCATGCTGACTCCGACCGA	*Dactylosporangium* sp.
*p4hD* -pEC-XK99E-A	GATCTCTAGATTAAACTGGCTGGGCAAG	

### Media

Luria broth (LB) medium, tryptone 10 g/L; yeast extract 5 g/L; solium chloride 10 g/L, was used for seed cultivation of *E. coli* strains. LBG medium containing 1% glucose additionally was used for *C. glutamicum* seed cultivation.

The medium (MEC) for batch culture of *E. coli* in shake flasks contained: glucose 10 g/L, glycerol 5 g/L, CO(NH_2_)_2_ 10 g/L, yeast extract 10 g/L, K_2_HPO_4_ 1 g/L, NaCl 2 g/L, MgSO_4_ · 7H_2_0 0.2 g/L, FeSO_4_ · 7H_2_O 1 mM, MnSO_4_ · 4H_2_O 10 mg/L, ZnSO_4_ · 7H_2_O 10 mg/L, VB_1_ 200 ug/L.

The medium (MCG) for batch culture of *C. glutamicum* in shake flasks contained: glucose 10 g/L; glycerol 5 g/L; CO(NH_2_)_2_ 10 g/L; corn syrup 15 g/L; K_2_HPO_4_ 1 g/L; NaCl 2 g/L; MgSO_4_ · 7H_2_0 0.2 g/L; FeSO_4_ · 7H_2_O 1 mM; MnSO_4_ · 4H_2_O 10 mg/L; ZnSO_4_ · 7H_2_O 10 mg/L; VB_1_ 200 mg/L; ethyl alcohol absolute 1.5%.

### Cultivations

The seed culture of *E. coli* strains was prepared by transferring 1 ml of glycerol stock to 30 ml of LB medium in a 250-ml flask, which was incubated overnight at 37°C and 220 rpm. Then 6% of seed culture was inoculated into 30 ml of MEC medium in a 250 ml flask and incubated at 37°C and 220 rpm for about 36 h. The initial pH of the medium was adjusted to 7.4. Induction (0.5 mM IPTG) was performed when the optical density was around 0.5, and the growth temperature was reduced from initial 37°C to 30°C. The cultivation of *C. glutamicum* was similar to that of *E. coli* expect that they were conducted at 30°C in the whole process using MCG medium. Experiments were performed in parallel on the same media without any induction.

### *Trans*-P4H activities

The intracellelular *trans-*P4H activities were measured by the whole-cell reaction procedures. After 8 hours induction in fermentation medium, cells were harvested by centrifugation at 12000× g for 20 min. The harvested cells were resuspended in the reaction mixture as followed. Each reaction mixture contained 80 mM 2-[N- morpholino] ethanesulfonic acid (MES) buffer (pH 6.5), 4 mM L-proline, 8 mM 2 - ketoglutarate, 2 mM FeSO4, 4 mM L-ascorbic acid The final cell concentration was about 100 g wet weight/L. The reaction mixtures were incubated at 35°C for 10 min and then cellular activity was inactivated completely by heat treatment at 100°C and 5 minutes. The amount of *trans-*4-hydroxy-L-proline in the supernatant of each mixture after centrifugation was determined. The amount of the enzyme which forms 1 nmol of Hyp in one minute was defined as 1 U.

### Analytical methods

The cell concentration was determined by measuring the optical density of appropriately diluted sample at 600 nm (*E. coli*, OD_600_) and 620 nm (*C. glutamicum* OD_620_) with a UV-visible spectroscopy system (Xinmao, Shanghai, China). Hyp was oxidized by Chloramine T and analyzed using spectrophotometric determination [35].

### Statistical analysis

All measurements for growth, *trans-*Hyp production and *trans-*P4H activity were performed in triplicate, and the data were averaged and presented as the mean ± standard deviation.

## Competing interests

The authors declare that they have no competing interests.

## Authors’ contributions

YY participated in the design and conducted the experiments in the study, and drafted the manuscript. HS participated in the analysis of proline and hydroxyproline. ZL conceived the study and helped to draft the manuscript. QY discussed the experiments and manuscript. All authors read and approved the final manuscript.

## Supplementary Material

Additional file 1 Supplement. Click here for file
