# Supplementary material for: Biosynthesis of trans-4-hydroxyproline by recombinant strains of Corynebacterium glutamicum and Escherichia coli
Source: BMC Biotechnol. 2014 May 19;14:44. doi: 10.1186/1472-6750-14-44 (PMC4055215; doi:10.1186/1472-6750-14-44)
Supplement: Additional file 1 — Supplement. [file 1472-6750-14-44-S1.doc]

**Supplement**

ATGCTGACCCCGACGGAGCTCAAGCAGTACCGCGAGGCGGGCTATCTGCTCATCGAGGAC 60

ATGCTGACTCCGACCGAGTTGAAGCAGTACCGTGAAGCAGGTTACTTGTTGATCGAGGAT 60

GGCCTCGGCCCGCGGGAGGTCGACTGCCTGCGCCGGGCGGCGGCGGCCCTCTACGCGCAG 120

GGTCTGGGACCGCGTGAGGTGGACTGCCTGCGACGCGCGGCTGCCGCACTCTACGCACAA 120

GACTCGCCGGACCGCACGCTGGAGAAGGACGGCCGCACCGTGCGCGCGGTCCACGGCTGC 180

GACTCACCCGACCGAACGCTCGAGAAAGATGGCCGCACCGTACGCGCGGTCCACGGCTGC 180

CACCGGCGCGACCCGGTCTGCCGCGACCTGGTCCGCCACCCGCGCCTGCTGGGCCCGGCG 240

CATCGTCGCGATCCCGTCTGTCGCGACCTCGTCCGTCACCCTCGTCTGCTTGGTCCAGCAG 240

ATGCAGATCCTGTCCGGCGACGTGTACGTCCACCAGTTCAAGATCAACGCGAAGGCCCCG 300

ATGCAGATTCTCTCGGGTGACGTGTATGTGCACCAATTCAAAATCAACGCAAAGGCCCCG 300

ATGACCGGCGATGTCTGGCCGTGGCACCAGGACTACATCTTCTGGGCCCGAGAGGACGGC 360

ATGACCGGTGACGTTTGGCCTTGGCATCAGGACTACATTTTCTGGGCTCGCGAAGATGGT 360

ATGGACCGTCCGCACGTGGTCAACGTCGCGGTCCTGCTCGACGAGGCCACCCACCTCAAC 420

ATGGATCGCCCCCACGTCGTCAACGTTGCTGTGCTGCTTGATGAAGCTACGCACCTGAAC 420

GGGCCGCTGTTGTTCGTGCCGGGCACCCACGAGCTGGGCCTCATCGACGTGGAGCGCCGC 480 GGCCCACTCCTGTTTGTGCCCGGTACCCATGAACTCGGCCTGATCGATGTTGAACGTCGC 480

GCGCCGGCCGGCGACGGCGACGCGCAGTGGCTGCCGCAGCTCAGCGCCGACCTCGACTAC 540

GCACCAGCGGGCGATGGCGATGCGCAATGGCTGCCTCAGTTGAGCGCTGATCTGGATTAC 540

GCCATCGACGCCGACCTGCTGGCCCGGCTGACGGCCGGGCGGGGCATCGAGTCGGCCACC 600

GCTATTGACGCAGATTTGCTGGCCCGCCTGACAGCCGGCCGCGGCATCGAATCCGCAACC 600

GGCCCGGCGGGCTCGATCCTGCTGTTCGACTCCCGGATCGTGCACGGCTCGGGCACGAAC 660

GGCCCAGCGGGATCCATCCTGCTCTTCGACTCTCGAATCGTTCACGGTTCCGGCACTAAC 660

ATGTCGCCGCACCCGCGCGGCGTCGTCCTGGTCACCTACAACCGCACCGACAACGCCCTG 720

ATGTCCCCGCACCCTCGCGGAGTAGTGCTGGTCACTTATAATCGTACCGACAATGCGCTG 720

CCGGCGCAGGCCGCTCCGCGCCCGGAGTTCCTGGCCGCCCGCGACGCCACCCCGCTGGTG 780

CCAGCCCAAGCTGCTCCCCGCCCAGAATTCCTCGCAGCCCGCGATGCCACCCCGCTTGTG 780

CCGCTGCCCGCGGGCTTCGCGCTGGCCCAGCCCGTCTAG 819

CCACTCCCTGCGGGATTTGCACTTGCCCAGCCAGTTTAA 819
